# Periapical Lesions in Panoramic Radiography and CBCT Imaging—Assessment of AI’s Diagnostic Accuracy

**DOI:** 10.3390/jcm13092709

**Published:** 2024-05-04

**Authors:** Wojciech Kazimierczak, Róża Wajer, Adrian Wajer, Veronica Kiian, Anna Kloska, Natalia Kazimierczak, Joanna Janiszewska-Olszowska, Zbigniew Serafin

**Affiliations:** 1 Department of Radiology and Diagnostic Imaging, Collegium Medicum, Nicolaus Copernicus University in Torun, Jagiellońska 13-15, 85-067 Bydgoszcz, Poland; 2 Department of Radiology and Diagnostic Imaging, University Hospital no 1 in Bydgoszcz, Marii Skłodowskiej Curie 9, 85-094 Bydgoszcz, Poland; 3 Kazimierczak Private Medical Practice, Dworcowa 13/u6a, 85-009 Bydgoszcz, Poland; 4 Dental Primus, Poznańska 18, 88-100 Inowrocław, Poland; 5 The Faculty of Medicine, Bydgoszcz University of Science and Technology, Kaliskiego 7, 85-796 Bydgoszcz, Poland; 6 Department of Interdisciplinary Dentistry, Pomeranian Medical University in Szczecin, Al. Powstańców Wlkp. 72, 70-111 Szczecin, Poland

**Keywords:** artificial intelligence (AI), automatic detection, cone-beam computed tomography, diagnosis, diagnostic test accuracy, orthopantomograms, panoramic radiograph, periapical lesion, periapical periodontitis

## Abstract

**Background/Objectives**: Periapical lesions (PLs) are frequently detected in dental radiology. Accurate diagnosis of these lesions is essential for proper treatment planning. Imaging techniques such as orthopantomogram (OPG) and cone-beam CT (CBCT) imaging are used to identify PLs. The aim of this study was to assess the diagnostic accuracy of artificial intelligence (AI) software Diagnocat for PL detection in OPG and CBCT images. **Methods**: The study included 49 patients, totaling 1223 teeth. Both OPG and CBCT images were analyzed by AI software and by three experienced clinicians. All the images were obtained in one patient cohort, and findings were compared to the consensus of human readers using CBCT. The AI’s diagnostic accuracy was compared to a reference method, calculating sensitivity, specificity, accuracy, positive predictive value (PPV), negative predictive value (NPV), and F1 score. **Results**: The AI’s sensitivity for OPG images was 33.33% with an F1 score of 32.73%. For CBCT images, the AI’s sensitivity was 77.78% with an F1 score of 84.00%. The AI’s specificity was over 98% for both OPG and CBCT images. **Conclusions**: The AI demonstrated high sensitivity and high specificity in detecting PLs in CBCT images but lower sensitivity in OPG images.

## 1. Introduction

Periapical lesions (PLs) are among the most common dental pathologies, the prevalence of which is estimated to be 52% at the individual level and 5% at the tooth level [1]. PLs are manifestations of apical periodontitis (AP) and are defined as barriers that restrict inflammation related to pathogens and their toxins in root canals [2,3]. PLs present as periapical radiolucencies and are usually asymptomatic, often being incidental radiographic findings [4]. PLs both impact the survival of affected teeth and have the potential to seriously compromise systemic health [5,6]. The most prevalent complication of persistent AP is tooth loss. The presence of these lesion also affects clinical decision making in various medical interventions, from observation to prosthetic treatment [3] and cardiac surgery [7].

Periapical radiolucencies should not be considered the sole manifestation of AP and might also be attributed to non-inflammatory processes. Since inflammatory processes account for 78% of periapical radiolucencies, not all lesions near the tooth root are due to inflammation [8]. The differential diagnosis includes both odontogenic and non-odontogenic lesions, encompassing benign and malignant entities. Moreover, trauma can result in changes resembling a PL. Clinical assessment and pulp vitality tests remain the basic methods for assessing teeth and informing treatment decisions [9].

Radiological examination is a fundamental tool in patient management. Dental diagnostics and treatment planning, in addition to monitoring treatment outcomes and complications, rely on both clinical examination and diagnostic imaging [10]. The primary diagnostic methods used in dental diagnostics include periapical radiographs (PRs), OPGs, and CBCT images [3]. PRs are the most widely utilized modality in dental lesion detection, boasting a high diagnostic accuracy exceeding 90% [3,11]. An OPG is a basic imaging modality that often serves as a first-line diagnostic tool for evaluating both the mandible and maxilla using one exposure. The diagnostic accuracy of OPGs in PL detection remains low, with a sensitivity ranging from 28% to 48.8% [12,13,14,15]. However, OPG imaging has high specificity and positive predictive value (PPV) for detecting PLs [14], and its diagnostic accuracy relies heavily on the location of the lesion [15]. PRs and OPGs are both associated with inherent limitations, such as the superimposition of anatomical structures, geometrical distortion, anatomical noise, and a two-dimensional representation [10]. Moreover, the PL must reach 30–50% bone mineral loss to become radiographically visible [16].

Since its introduction in the 2000s, CBCT has proven to be a valuable tool for endodontic assessment, as validated in many studies [10,17,18,19]. CBCT overcomes the limitations of conventional two-dimensional dental imaging by providing accurate insights into the multiplanar details of dental and bony structures with a spatial resolution of less than 100 µm [20]. In the diagnosis of PLs, CBCT has been shown to be more effective than periapical radiography (PR) in terms of intra- and interobserver agreement, communication with patients and other practitioners, and outcome assessment [10,21]. Furthermore, multiple studies have confirmed that CBCT offers higher diagnostic accuracy in detecting PL, with approximately one-third of lesions being missed by PR [22,23,24]. A recent study by Mostafapoor et al. [25] demonstrated a 95% sensitivity and 90% specificity for CBCT in the diagnosis of PL. However, there are some conflicting results showing possible overdiagnosis of PL, confirmed by negative results from a histological analysis [9,26].

The recent boom in the utilization of artificial intelligence (AI) tools in medicine has not bypassed dentistry, finding particular relevance in the field of dentomaxillofacial radiology [27,28,29]. The ever-increasing number of radiological examinations [30], coupled with the increasing work burden on practitioners, has spurred the development of tools to facilitate radiological diagnostics. A system developed by Diagnocat Ltd. (San Francisco, CA, USA), which utilizes a convolutional neural network (CNN), aims to provide precise and comprehensive dental diagnostics. The company claims that the system was trained on over 35,000 dental radiographs to ensure its diagnostic performance. One of the systems’ distinctive features is the use of a PL detection tool, which aids in prompt diagnosis. Despite promising results [31,32,33], some authors have reported the unacceptably low accuracy of AI in PL assessment via orthopantomograms (OPGs) [34]. Therefore, evaluating the diagnostic accuracy of AI for detecting PL via both OPG and CBCT is pertinent.

The aim of the present study was to compare the diagnostic performance of an AI-driven platform for detecting periapical lesions in OPG and CBCT images acquired from the same patients and to compare the program’s results with those of an experienced human reader’s evaluation.

## 2. Materials and Methods

### 2.1. Patients

The population of this retrospective diagnostic accuracy study initially consisted of 92 consecutive patients referred for OPG and CBCT imaging at a private dental center. All patients were referred to both imaging modalities by orthodontists or dental surgeons between January and September 2023. After the initial diagnostic OPGs were obtained, the selected patients were referred for CBCT scans. The primary clinical indications necessitating CBCT scans were suspicions of PLs in the OPGs or the presence of an impacted tooth. A large FOV (10 × 13 cm) was used in all cases with suspicion of PLs and/or the presence of unerupted teeth on both sides of the dental arch. The main inclusion criterion for this study was the availability of both OPG and CBCT images taken within a 30-day interval to minimize the impact of dental procedures, aging, and other factors on dental status. The exclusion criteria consisted of the presence of a study range not covering periapical regions of all the present teeth, severe motion artifacts, and poor overall image quality. After the initial selection of patients with both OPG and CBCT images, 43 patients were excluded due to the use of a small FOV not covering the periapical regions of all the present teeth. The authors reviewed the CBCT scans and OPGs of 55 patients. Six of these patients were excluded because they did not meet the inclusion criterion due to the presence of motion artifacts and poor image quality. After applying the eligibility criteria, 49 patients were selected for the final study group from the initial group of 92 patients with both OPGs and CBCT images.

The sample size was calculated with the formula presented by Buderer for diagnostic accuracy studies [35]. The following assumptions were made: expected sensitivity, 85%; expected specificity, 95%; prevalence of the disease, 70%; precision, 0.1; and confidence level, 90%. Moreover, a web-based sample size calculator was used (https://wnarifin.github.io/ssc/sssnsp.html (accessed on 30 April 2024)).

### 2.2. Image Acquisition and Post-Processing

All the CBCT and OPG images were acquired using a Hyperion X9 PRO 13 × 16 (MyRay, Imola, Italy) machine. One standard, marked as the “Regular” setting of the apparatus, was used (90 KV, 36 mAs, CTDI/Vol 4.09 mGy, and 13 cm field of view (FOV)) in the CBCT scans. All CBCT images were reconstructed with a slice thickness of 0.3 mm. Patient identifiers were removed to maintain anonymity, and images were coded for blinded analysis.

The reading sessions were performed on a dedicated console, using iRYS Viewer software version 6.2 (MyRay, Italy) software. During the CBCT analysis, the viewer’s window width and center were predefined at 1048 and 4096 HU, respectively. All images were evaluated using a RadiForce MX243W monitor (Eizo, Hakusan, Japan), which is certified for medical use. The reading sessions were conducted in a CT reporting room without access to sunlight and with dimmed light to ensure appropriate conditions for the evaluation of radiological examinations.

### 2.3. Multireader Evaluation

The images were independently evaluated by two readers: one orthodontist and one radiologist, both with more than 8 years of experience. The presence or absence of PLs was recorded for each tooth. The readers assessed the presence of PLs based on signs of bone destruction. Each reader assessed each radiograph separately and independently (without knowledge of the AI results or the other reader’s evaluation). The readers initially evaluated the OPGs, followed by the CBCT images. The OPG and CBCT evaluation sessions were conducted at least one month apart to avoid potential bias from the OPG reading session. After the evaluation, the readers discussed the results, jointly evaluated images, and reached a consensus, which was considered the reference standard. To assess the reliability of the AI diagnostic reports, they were compared with the consensus reached by the readers.

### 2.4. AI Evaluation

Both sets of images (OPG + CBCT) of each included patient were manually uploaded to the cloud-based, commercially available platform Diagnocat (Diagnocat Ltd., San Francisco, CA, USA). The AI software automatically provided separate reports for both imaging modalities with estimated probabilities of the lesion occurring. The program’s threshold for a positive diagnosis was the calculated probability of lesion occurrence higher than 50%.

### 2.5. Statistical Evaluation

The diagnostic performance of the AI program was assessed in comparison to that of the common reference method. The sensitivity, specificity, accuracy, positive predictive value (PPV), negative predictive value (NPV), and F1 score were calculated. The formulas used in diagnostic performance calculations and further explanations can be found in Hicks et al. [36]. The significance level was set to 0.05. All analyses were conducted in R software, version 4.3.2.

## 3. Results

### 3.1. Population

The study material consisted of 49 sets of images. The mean age of all participants was 41 years (range 12–70). A total of 37 females with a mean age of 42.11 years (SD 13.39; range, 12–65 years) and 12 males with a mean age of 51.75 years (SD 12.46; range, 31–70 years) were included.

The results of sample size calculations showed that our group was sufficient for diagnostic accuracy calculations.

### 3.2. Diagnostic Accuracy

A summary of the detected PLs and the number of analyzed teeth is available in Table 1.

The analyzed AI software presented low (33.33%) sensitivity in the detection of PLs in OPGs compared to the consensus of human readers (CBCT analysis).

**Figure 1:**
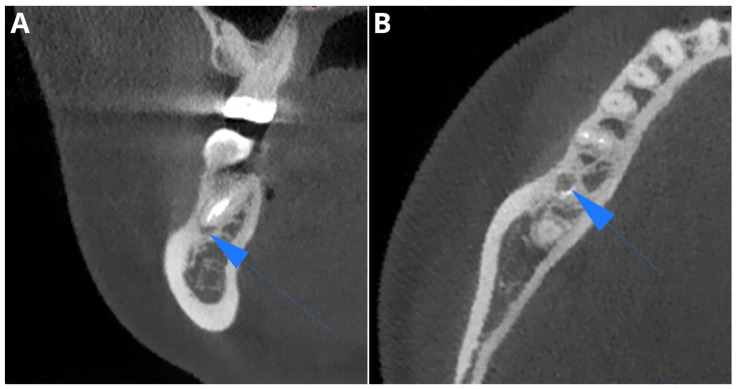
One of the cases misdiagnosed by the AI tool as a widened PDL space but classified as PL by human readers (blue arrows). Coronal plane (**A**), axial plane (**B**).

The calculated sensitivity of the AI CBCT reports for PL detection was 77.78%. A summary of the diagnostic accuracy parameters is available in Table 2. In total, the analyzed software showed four false negative results and one false positive result in the CBCT assessment. Figure 1 presents one of the false negative cases misdiagnosed by Diagnocat as a widened periodontal ligament (PDL) space but classified as PL by human readers.

**Figure 2:**
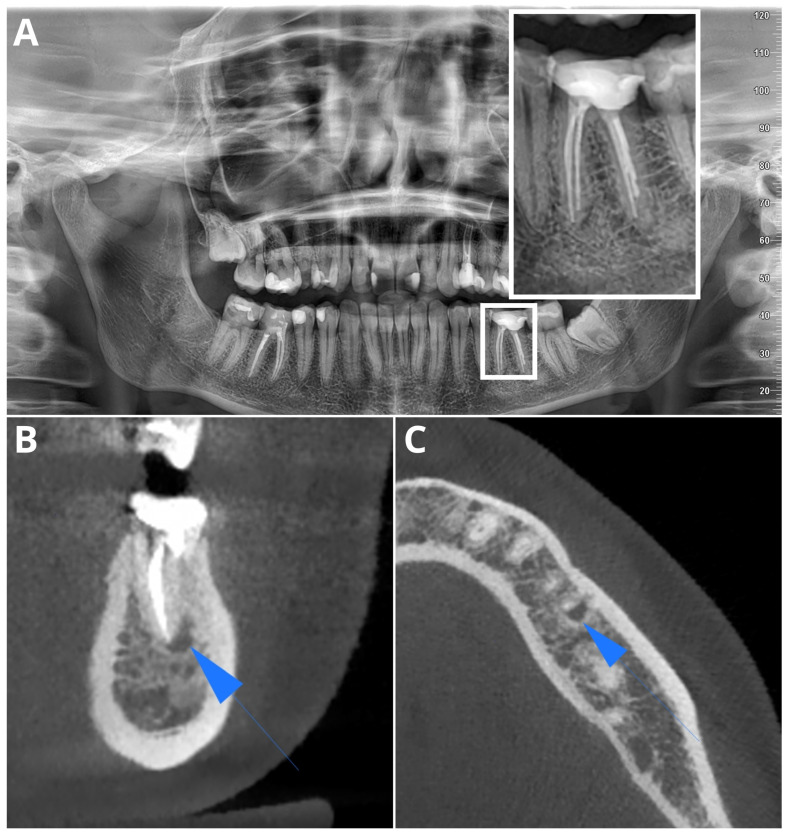
Sample false negative AI diagnosis in OPG, diagnosed by readers in OPG (**A**) (lesion magnified in white rectangle). PL confirmed in CBCT (blue arrows): coronal plane (**B**), axial plane (**C**).

**Figure 3:**
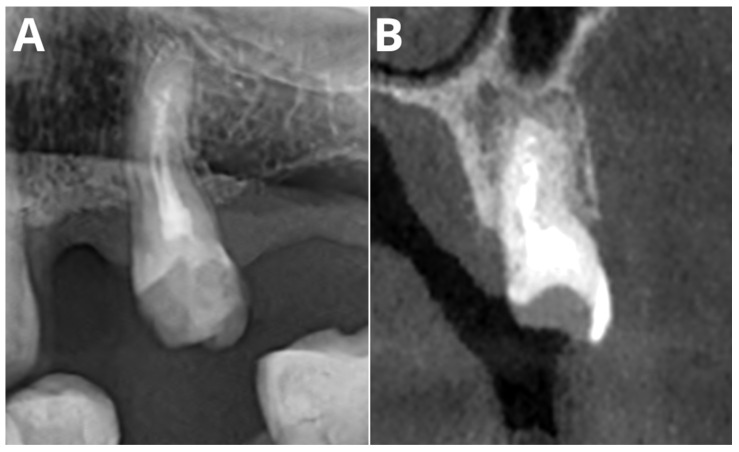
Sample false positive AI diagnosis of PL in OPG, correctly diagnosed by readers in OPG (**A**). No signs of periapical radiolucency in CBCT (**B**).

Figure 2 and Figure 3 present, respectively, a false negative and a false positive AI OPG diagnosis.

**Figure 4:**
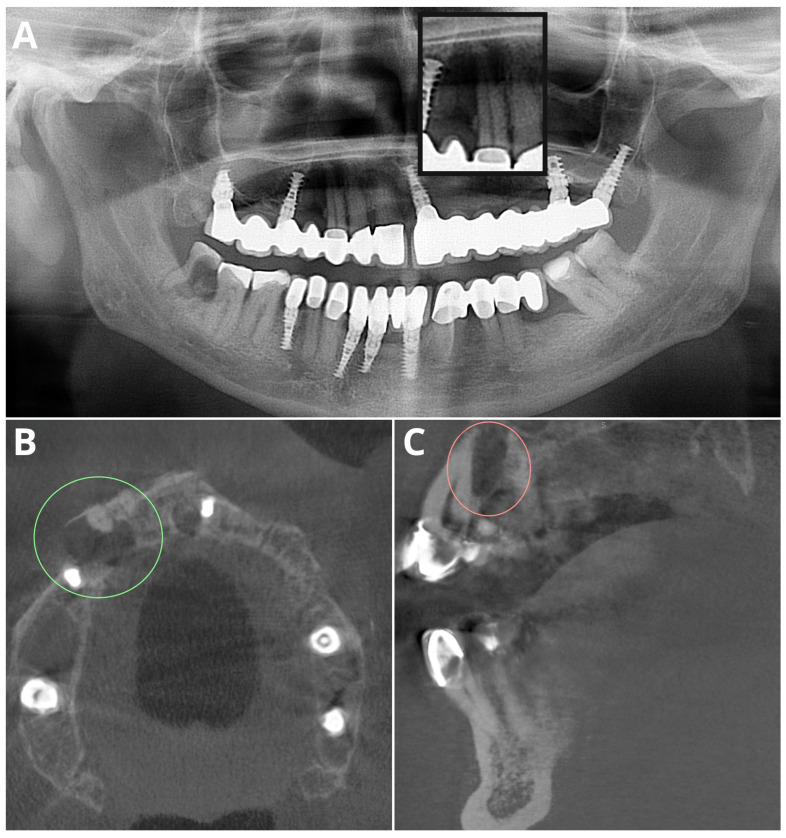
Sample false negative AI diagnosis in both OPG and CBCT evaluations, caused by high image noise from metal artifacts. In the OPG image, the lesion is magnified in the black rectangle (**A**). CBCT images of PL in color ovals in the axial plane (**B**) and sagittal plane (**C**).

All of the analyzed modalities showed very high (>95%) specificity. An example of a false negative AI diagnosis in both OPG and CBCT is shown in Figure 4.

The analysis readers’ OPG assessments showed very interesting findings, with a sensitivity of 66.67% (compared to readers’ consensus on CBCT analysis). However, there was a high number of false positives, resulting in a low positive predictive value (PPV) of 31.58% (Table 3).

The analysis of the AI’s OPG assessment results when compared to the readers’ consensus on the OPG assessment showed a very low sensitivity of 21.05% paired with a low F1 score of 28.24% (Table 4). However, these results are mainly attributed to the high number of false positives in the readers’ OPG analysis.

## 4. Discussion

Our study aimed to assess the diagnostic accuracy of an AI-driven platform for detecting PLs in OPGs and CBCT images. The results showed that the AI tool had high specificity and PPV in both the OPG and CBCT analyses. However, the sensitivity of the AI tool varies depending on the modality. According to the OPG analysis, the AI tool had a sensitivity of 33.33% compared to the reference standard, while according to the CBCT analysis, it had a sensitivity of 77.78%. These findings indicate that the AI tool has limitations in accurately detecting PLs in OPGs but has a relatively high diagnostic performance in CBCT evaluation for PL detection. Additionally, our results showed that OPG analysis is not a reliable method for assessing PLs, with high variability in the results of both the AI’s and human readers’ analyses compared to the reference standard (CBCT assessment).

PR remains the first-choice modality for PL assessment due to its superior resolution, accessibility, and low radiation dose [37]. Recent advancements in AI technology have led to significant progress in developing algorithms capable of detecting PLs and caries [38,39]. The assessment of periapical radiographs using AI has shown promise in enhancing diagnostic accuracy and improving clinicians’ performance in PL detection [40,41,42]. However, the performance of AI on OPGs presents additional challenges. Panoramic imaging is inherently prone to image distortion, overlap, and lower resolution compared to PR, which can hinder AI’s ability to accurately diagnose PLs. Other limiting factors include low contrast and unclear contours of the teeth [43]. Furthermore, the sensitivity of PL detection heavily relies on the size of the lesion. A study by Nardi et al. [12] showed that the sensitivity of OPG for detecting PLs larger than 4.6 mm is 48.5%, but when the lesion is smaller than 4.5 mm, the sensitivity sharply drops to 20.0%. The authors concluded that such small lesions do not represent 30–50% of mineral bone loss, which is considered the threshold in the radiographic detection of PL. These limitations make the accurate detection of PLs in OPGs challenging and have influenced the results of our analysis.

The findings from the analysis of the readers’ OPG assessment reveal a moderate level of sensitivity, meaning that the readers were able to correctly identify 66.67% of the positive cases compared to the readers’ consensus on CBCT analysis. However, there is a high number of false positives, resulting in a low positive predictive value (PPV) of 31.58%. This suggests that while the readers are good at detecting positive cases, they also incorrectly identify a significant number of cases as positive. On the other hand, the analysis of the AI OPG assessment shows a very low sensitivity of 21.05% compared to OPG readers’ consensus, indicating that the AI system is not effective at correctly identifying positive cases compared to the readers’ consensus on OPG analysis. However, it is worth noting that these results are largely affected by the high number of false positives found in the readers’ OPG analysis. In combination, these results highlight the high variability of OPG-based PL assessments and indicate that OPGs are not the preferable imaging modality in this task.

Similarly, the low sensitivity of OPG for PL detection, as shown in our study, has already been reported in the literature [12,13,14,15]. An interesting study by Zadrożny et al. [34] showed very similar results in terms of the use of OPG for PL detection. The study analyzed the performance of the Diagnocat platform and reported a sensitivity of 39.0% on OPGs when paired with a high specificity of 98.1%. The AI tool showed similar results in caries assessment (sensitivity of 44.5%, specificity of 98.2%). The authors concluded that the tested AI tool presented unacceptable diagnostic performance in detecting PLs and caries. Several other studies have assessed the diagnostic accuracy of OPGs/panoramic radiographs in PL assessment, showcasing various CNNs and U-Net architectures developed by the authors [44,45,46,47,48]. These studies reported highly variable sensitivity and F1 scores for PL detection. The deep learning (DL) algorithm developed by Endres et al. exhibited a low F1 score [48]. Nevertheless, the authors concluded that the trained algorithm outperformed 14 of the 24 oral-maxillofacial surgeons participating in their study, suggesting that the algorithm has the potential to assist surgeons in PL diagnostics. A similar study by Çelik et al. [47] demonstrated the high diagnostic performance of DL in PL detection, with F1 scores ranging between 0.8 and 0.895. Another paper by Bayrakdar et al. [46] presented impressive diagnostic performance rates for a tested U-Net, with sensitivity, precision, and an F1 score for the segmentation of PLs of 0.92, 0.84, and 0.88, respectively. Song’s results were also strong; although, the F1 scores were slightly lower (74.2–82.8%) [45]. In our view, this indicates significant differences in the performance of AI models tested. The quality of the images analyzed also undoubtedly plays a role. We believe that a highly reliable evaluation of the selected AI tools’ capabilities would be possible in studies evaluating publicly available radiological datasets. Diagnocat analyzes a diverse array of data sent from around the world, in various formats and of varying quality. Therefore, it is conceivable that the excellent results achieved by experimental AI algorithms in laboratory settings might not be reproducible when applied to data from sources other than those used for training [49].

CBCT overcomes the limitations of OPG imaging by providing accurate insights into the multiplanar details of the tooth and bony structures. The higher sensitivity of the AI tool in CBCT analyses can be attributed to the superior imaging capabilities of CBCT compared to those of OPG. Our study supports the thesis of high AI performance in CBCT evaluation. The reported sensitivity of PL detection is in line with that of other studies [31,50,51,52,53]. A study by Orhan et al. [31] showed that Diagnocat achieved 92.8% accuracy in PL detection on CBCT images. Similar results were presented in a 2020 study by Brignardello [53], in which 93% sensitivity and no significant differences were detected in the volumetric assessment of PLs. Comparable results of different CNN networks in CBCT PL detection were subsequently achieved by several authors [54,55,56,57]. Figure 3 depicts one of the false negative diagnoses made by the AI in a CBCT image. In our opinion, this was most likely due to the high noise level in the image and the presence of metallic artifacts. This indicates a direction for further improvements in future versions of the AI tool.

To date, few meta-analyses and systematic reviews have been conducted on the utilization of AI for PL detection [58,59,60,61,62,63,64]. An important study by Silva et al. [60] showed that the pooled diagnostic accuracy of CBCT for PL detection was 88.75% (95% confidence interval = 85.19–92.30). However, this review included only four studies. In a 2023 review by Sadr et al. [62], the pooled sensitivity and specificity of the 28 included studies were 0.925 and 0.852, respectively. The conclusion was that AI exhibits high diagnostic accuracy for PL detection.

AI tools have also been employed in more comprehensive assessments. The 2021 study by Ezhov showed [65] that the Diagnocat AI platform significantly improved the diagnostic capabilities of the readers in numerous tasks (e.g., detection of periodontitis, PLs, and caries). In a comparison of the AI-aided and unaided groups, the pooled overall sensitivity calculations were 85.4% and 76.7%, respectively, while the specificity values were 96.7% and 96.2%, respectively. In our opinion, these results are promising, and the growing scientific evidence demonstrating the benefits of AI-aided, CBCT-based dental diagnostics will soon lead to the widespread use of AI tools in dental offices. With the increasing supply of AI tools and their mass utilization, prices are likely to decrease, and their availability will further increase, including for dental practices in developing countries.

However, a very important limitation of CBCT evaluation must be mentioned—although CBCT overcomes the limitations of conventional OPGs, concerns are raised about false positives in the diagnosis of PL. A retrospective study by Pope [9] revealed that 20% of vital teeth showed widening of the PDL on CBCT, potentially leading to overdiagnosis and overtreatment of early AP. However, this study’s methodology was questioned because of the use of symptoms as the only reference standard. Biopsies of periapical tissues are ideal for diagnosis but are impractical and unethical. Similar results were found in a study on persistent PLs post-surgery, where histology showed that 42% of the samples had no inflammation but that radiolucency was present on CBCT [26]. A correct diagnosis was reached in 63% of patients when radiolucency was evident in both the PR and CBCT images. The importance of PRs’ and CBCT’s tendency to overestimate the presence of radiolucency was emphasized [26].

The use of CBCT imaging must be carefully considered due to its primary drawback: the excessive radiation exposure. As stated in the joint position statement from the American Association of Endodontists and the American Academy of Oral and Maxillofacial Radiology, CBCT imaging should be reserved for cases where lower-dose conventional radiography or alternative imaging modalities fail to provide adequate diagnostic information [37]. Furthermore, it is generally true that a smaller FOV results in a lower radiation dose, while a smaller voxel size leads to higher resolution and reduced scatter and noise in CBCT images [66]. Therefore, in adherence to the ALARA (As Low As Reasonably Achievable) principles, CBCT is considered a secondary imaging option after intraoral radiography [37]. CBCT may be justified in instances where clinical findings and conventional PRs are inconclusive; however, its routine use in all cases of PL is unwarranted. Given that our study group was highly heterogeneous, the indications for large FOV CBCT imaging varied, including the presence of periapical lesions or impacted teeth on both sides of the dental arches, detection of bony changes, and pre-implant assessments. Under these circumstances, the use of CBCT appears justified.

Our study has the potential to stimulate further research comparing AI’s diagnostic accuracy in different radiological examinations within the same patient cohort. Diagnocat offers numerous, diverse modules which also demand further scientific evaluation—such as caries detection, endodontic treatment evaluation, and others. Subsequent studies evaluating the diagnostic parameters of these tools would be very interesting. Considering the continuous development of AI and promising directions for its application, such as in radiomics, we should expect that, in the future, AI may surpass clinicians in diagnostic accuracy. Further studies with larger and more diverse study groups will also allow for a more comprehensive assessment of the diagnostic value of AI.

The limitations of our study should be acknowledged. First, the study population was relatively small, which may limit the generalizability of the findings. Second, the AI tool used in the study was commercially available, and diagnostic performance may vary depending on the specific AI algorithm and training dataset used. Third, the study focused only on PL detection, and other dental pathologies were not considered. Fourth, only one CBCT/OPG apparatus was used. Fifth, due to geographical limitations, the study group was not ethnically diverse and included only white patients.

## 5. Conclusions

In conclusion, our study showed that an AI-driven platform had high specificity and PPV for detecting PLs in OPGs and CBCT images. The tested AI tool demonstrated relatively high sensitivity in CBCT analysis. However, the sensitivity of the AI tool was lower than that of human readers, especially in OPG analysis.

## Figures and Tables

**Table 1:** Summary of the findings of AI software and human readers (total number of teeth: 1223).

Parameter	AI Tool		Readers’ Consensus	
	OPG	CBCT	OPG	CBCT
PL	28	23	51	26

AI = artificial intelligence; OPG = orthopantomogram; CBCT = cone-beam computed tomography; PL = periapical lesion.

**Table 2:** Diagnostic accuracy parameters of the AI program’s OPG and CBCT analyses compared to reference standard (readers’ consensus on CBCT analysis).

Modality	Sensitivity	Specificity	Accuracy	PPV	NPV	F1
OPG	33.33%	98.43%	97.01%	32.14%	98.51%	32.73%
CBCT	77.78%	99.83%	99.35%	91.30%	99.50%	84.00%

PPV = positive predictive value; NPV = negative predictive value; AI = artificial intelligence; OPG = orthopantomogram; CBCT = cone-beam computed tomography.

**Table 3:** Diagnostic accuracy parameters of readers’ OPG analyses compared to reference standard (readers’ consensus on CBCT analysis).

Sensitivity	Specificity	Accuracy	PPV	NPV	F1
66.67%	96.82%	96.18%	31.58%	99.25%	42.86%

PPV = positive predictive value; NPV = negative predictive value.

**Table 4:** Diagnostic accuracy parameters of AI OPG analysis compared to the results of readers’ consensus on OPG analysis.

Sensitivity	Specificity	Accuracy	PPV	NPV	F1
21.05%	98.66%	95.13%	42.86%	96.33%	28.24%

PPV = positive predictive value; NPV = negative predictive value.

## Data Availability

Data are available on request.

## References

[1] Tibúrcio-Machado C.S., Michelon C., Zanatta F.B., Gomes M.S., Marin J.A., Bier C.A. (2021). The Global Prevalence of Apical Periodontitis: A Systematic Review and Meta-Analysis. Int. Endod. J..

[2] Sundqvist G. (1976). Bacteriological Studies of Necrotic Dental Pulps. Ph.D. Thesis.

[3] Karamifar K., Tondari A., Saghiri M.A. (2020). Endodontic Periapical Lesion: An Overview on the Etiology, Diagnosis and Current Treatment Modalities. Eur. Endod. J..

[4] Abbott P.V. (2004). Classification, Diagnosis and Clinical Manifestations of Apical Periodontitis. Endod. Topics.

[5] Kirkevang L.L., Ørstavik D., Bahrami G., Wenzel A., Væth M. (2017). Prediction of Periapical Status and Tooth Extraction. Int. Endod. J..

[6] Segura-Egea J.J., Martín-González J., Castellanos-Cosano L. (2015). Endodontic Medicine: Connections between Apical Periodontitis and Systemic Diseases. Int. Endod. J..

[7] Souza A.F., Rocha A.L., Castro W.H., Gelape C.L., Nunes M.C.P., Oliveira S.R., Travassos D.V., Silva T.A. (2017). Dental Management for Patients Undergoing Heart Valve Surgery. J. Card. Surg..

[8] Chapman M.N., Nadgir R.N., Akman A.S., Saito N., Sekiya K., Kaneda T., Sakai O. (2013). Periapical Lucency around the Tooth: Radiologic Evaluation and Differential Diagnosis. Radiographics.

[9] Pope O., Sathorn C., Parashos P. (2014). A Comparative Investigation of Cone-Beam Computed Tomography and Periapical Radiography in the Diagnosis of a Healthy Periapex. J. Endod..

[10] Patel S., Dawood A., Mannocci F., Wilson R., Pitt Ford T. (2009). Detection of Periapical Bone Defects in Human Jaws Using Cone Beam Computed Tomography and Intraoral Radiography. Int. Endod. J..

[11] Chang L., Umorin M., Augsburger R.A., Glickman G.N., Jalali P. (2020). Periradicular Lesions in Cancellous Bone Can Be Detected Radiographically. J. Endod..

[12] Nardi C., Calistri L., Pradella S., Desideri I., Lorini C., Colagrande S. (2017). Accuracy of Orthopantomography for Apical Periodontitis without Endodontic Treatment. J. Endod..

[13] Estrela C., Bueno M.R., Leles C.R., Azevedo B., Azevedo J.R. (2008). Accuracy of Cone Beam Computed Tomography and Panoramic and Periapical Radiography for Detection of Apical Periodontitis. J. Endod..

[14] Nardi C., Calistri L., Grazzini G., Desideri I., Lorini C., Occhipinti M., Mungai F., Colagrande S. (2018). Is Panoramic Radiography an Accurate Imaging Technique for the Detection of Endodontically Treated Asymptomatic Apical Periodontitis?. J. Endod..

[15] Nardi C., Calistri L., Pietragalla M., Vignoli C., Lorini C., Berti V., Mungai F., Colagrande S. (2020). Electronic Processing of Digital Panoramic Radiography for the Detection of Apical Periodontitis. Radiologia Medica.

[16] Bender I.B., Seltzer S. (2003). Roentgenographic and Direct Observation of Experimental Lesions in Bone: I. J. Endod..

[17] Arias E., Huang Y.H., Zhao L., Seelaus R., Patel P., Cohen M. (2019). Virtual Surgical Planning and Three-Dimensional Printed Guide for Soft Tissue Correction in Facial Asymmetry. J. Craniofacial Surg..

[18] Lo R.C., Buck D.B., Herrmann J., Hamdan A.D., Wyers M., Patel V.I., Fillinger M., Schermerhorn M.L. (2016). Risk factors and consequences of persistent type II endoleaks. J. Vasc. Surg..

[19] Garlapati K., Gandhi Babu D.B., Chaitanya N.C.S.K., Guduru H., Rembers A., Soni P. (2017). Evaluation of Preference and Purpose of Utilisation of Cone Beam Computed Tomography (CBCT) Compared to Orthopantomogram (OPG) by Dental Practitioners—A Cross-Sectional Study. Pol. J. Radiol..

[20] Kaasalainen T., Ekholm M., Siiskonen T., Kortesniemi M. (2021). Dental Cone Beam CT: An Updated Review. Phys. Medica.

[21] Barnett C.W., Glickman G.N., Umorin M., Jalali P. (2018). Interobserver and Intraobserver Reliability of Cone-Beam Computed Tomography in Identification of Apical Periodontitis. J. Endod..

[22] Davies A., Mannocci F., Mitchell P., Andiappan M., Patel S. (2015). The Detection of Periapical Pathoses in Root Filled Teeth Using Single and Parallax Periapical Radiographs versus Cone Beam Computed Tomography—A Clinical Study. Int. Endod. J..

[23] Lofthag-Hansen S., Huumonen S., Gröndahl K., Gröndahl H.G. (2007). Limited Cone-Beam CT and Intraoral Radiography for the Diagnosis of Periapical Pathology. Oral. Surg. Oral. Med. Oral. Pathol. Oral. Radiol. Endod..

[24] Abella F., Patel S., Duran-Sindreu F., Mercadé M., Bueno R., Roig M. (2012). Evaluating the Periapical Status of Teeth with Irreversible Pulpitis by Using Cone-Beam Computed Tomography Scanning and Periapical Radiographs. J. Endod..

[25] Mostafapoor M., Hemmatian S. (2022). Evaluation of the Accuracy Values of Cone-Beam CT Regarding Apical Periodontitis: A Systematic Review and Meta-Analysis. Oral. Radiol..

[26] Kruse C., Spin-Neto R., Reibel J., Wenzel A., Kirkevang L.L. (2017). Diagnostic Validity of Periapical Radiography and CBct for Assessing Periapical Lesions That Persist after Endodontic Surgery. Dentomaxillofacial Radiol..

[27] Heo M.S., Kim J.E., Hwang J.J., Han S.S., Kim J.S., Yi W.J., Park I.W. (2020). Dmfr 50th Anniversary: Review Article Artificial Intelligence in Oral and Maxillofacial Radiology: What Is Currently Possible?. Dentomaxillofacial Radiol..

[28] Kazimierczak N., Kazimierczak W., Serafin Z., Nowicki P., Nożewski J., Janiszewska-Olszowska J. (2024). AI in Orthodontics: Revolutionizing Diagnostics and Treatment Planning—A Comprehensive Review. J. Clin. Med..

[29] Abesi F., Jamali A.S., Zamani M. (2023). Accuracy of Artificial Intelligence in the Detection and Segmentation of Oral and Maxillofacial Structures Using Cone-Beam Computed Tomography Images: A Systematic Review and Meta-Analysis. Pol. J. Radiol..

[30] Hajem S., Brogårdh-Roth S., Nilsson M., Hellén-Halme K. (2020). CBCT of Swedish Children and Adolescents at an Oral and Maxillofacial Radiology Department. A Survey of Requests and Indications. Acta Odontol. Scand..

[31] Orhan K., Bayrakdar I.S., Ezhov M., Kravtsov A., Özyürek T. (2020). Evaluation of Artificial Intelligence for Detecting Periapical Pathosis on Cone-Beam Computed Tomography Scans. Int. Endod. J..

[32] Issa J., Jaber M., Rifai I., Mozdziak P., Kempisty B., Dyszkiewicz-Konwińska M. (2023). Diagnostic Test Accuracy of Artificial Intelligence in Detecting Periapical Periodontitis on Two-Dimensional Radiographs: A Retrospective Study and Literature Review. Medicina.

[33] Vujanovic T., Jagtap R. (2023). Evaluation of Artificial Intelligence for Automatic Tooth and Periapical Pathosis Detection on Panoramic Radiography. Oral. Surg. Oral. Med. Oral. Pathol. Oral. Radiol..

[34] Zadrożny Ł., Regulski P., Brus-Sawczuk K., Czajkowska M., Parkanyi L., Ganz S., Mijiritsky E. (2022). Artificial Intelligence Application in Assessment of Panoramic Radiographs. Diagnostics.

[35] Fenn Buderer N.M. (1996). Statistical Methodology: I. Incorporating the Prevalence of Disease into the Sample Size Calculation for Sensitivity and Specificity. Acad. Emerg. Med..

[36] Hicks S.A., Strümke I., Thambawita V., Hammou M., Riegler M.A., Halvorsen P., Parasa S. (2022). On Evaluation Metrics for Medical Applications of Artificial Intelligence. Sci. Rep..

[37] Fayad M.I., Nair M., Levin M.D., Benavides E., Rubinstein R.A., Barghan S., Hirschberg C.S., Ruprecht A. (2015). AAE and AAOMR Joint Position Statement Use of Cone Beam Computed Tomography in Endodontics 2015 Update. Oral Surg. Oral Med. Oral Pathol. Oral Radiol..

[38] Putra R.H., Doi C., Yoda N., Astuti E.R., Sasaki K. (2022). Current Applications and Development of Artificial Intelligence for Digital Dental Radiography. Dentomaxillofacial Radiol..

[39] Musri N., Christie B., Ichwan S.J.A., Cahyanto A. (2021). Deep Learning Convolutional Neural Network Algorithms for the Early Detection and Diagnosis of Dental Caries on Periapical Radiographs: A Systematic Review. Imaging Sci. Dent..

[40] Chen H., Li H., Zhao Y., Zhao J., Wang Y. (2021). Dental Disease Detection on Periapical Radiographs Based on Deep Convolutional Neural Networks. Int. J. Comput. Assist. Radiol. Surg..

[41] Pauwels R., Brasil D.M., Yamasaki M.C., Jacobs R., Bosmans H., Freitas D.Q., Haiter-Neto F. (2021). Artificial Intelligence for Detection of Periapical Lesions on Intraoral Radiographs: Comparison between Convolutional Neural Networks and Human Observers. Oral. Surg. Oral. Med. Oral. Pathol. Oral. Radiol..

[42] Hamdan M.H., Tuzova L., Mol A., Tawil P.Z., Tuzoff D., Tyndall D.A. (2022). The Effect of a Deep-Learning Tool on Dentists’ Performances in Detecting Apical Radiolucencies on Periapical Radiographs. Dentomaxillofacial Radiol..

[43] Lee J.H., Han S.S., Kim Y.H., Lee C., Kim I. (2020). Application of a Fully Deep Convolutional Neural Network to the Automation of Tooth Segmentation on Panoramic Radiographs. Oral Surg. Oral Med. Oral Pathol. Oral Radiol..

[44] İçöz D., Terzioǧlu H., Özel M.A., Karakurt R. (2023). Evaluation of an Artificial Intelligence System for the Diagnosis of Apical Periodontitis on Digital Panoramic Images. Niger. J. Clin. Pract..

[45] Song I.S., Shin H.K., Kang J.H., Kim J.E., Huh K.H., Yi W.J., Lee S.S., Heo M.S. (2022). Deep Learning-Based Apical Lesion Segmentation from Panoramic Radiographs. Imaging Sci. Dent..

[46] Bayrakdar I.S., Orhan K., Çelik Ö., Bilgir E., Saǧlam H., Kaplan F.A., Görür S.A., Odabaş A., Aslan A.F., Różyło-Kalinowska I. (2022). A U-Net Approach to Apical Lesion Segmentation on Panoramic Radiographs. Biomed. Res. Int..

[47] Çelik B., Savaştaer E.F., Kaya H.I., Çelik M.E. (2023). The Role of Deep Learning for Periapical Lesion Detection on Panoramic Radiographs. Dentomaxillofacial Radiol..

[48] Endres M.G., Hillen F., Salloumis M., Sedaghat A.R., Niehues S.M., Quatela O., Hanken H., Smeets R., Beck-Broichsitter B., Rendenbach C. (2020). Development of a Deep Learning Algorithm for Periapical Disease Detection in Dental Radiographs. Diagnostics.

[49] Waller J., O’connor A., Rafaat E., Amireh A., Dempsey J., Martin C., Umair M. (2022). Applications and Challenges of Artificial Intelligence in Diagnostic and Interventional Radiology. Pol. J. Radiol..

[50] Vinayahalingam S., Goey R.S., Kempers S., Schoep J., Cherici T., Moin D.A., Hanisch M. (2021). Automated Chart Filing on Panoramic Radiographs Using Deep Learning. J. Dent..

[51] Başaran M., Çelik Ö., Bayrakdar I.S., Bilgir E., Orhan K., Odabaş A., Aslan A.F., Jagtap R. (2022). Diagnostic Charting of Panoramic Radiography Using Deep-Learning Artificial Intelligence System. Oral. Radiol..

[52] Zhu J., Chen Z., Zhao J., Yu Y., Li X., Shi K., Zhang F., Yu F., Shi K., Sun Z. (2023). Artificial Intelligence in the Diagnosis of Dental Diseases on Panoramic Radiographs: A Preliminary Study. BMC Oral Health.

[53] Brignardello-Petersen R. (2020). Artificial Intelligence System Seems to Be Able to Detect a High Proportion of Periapical Lesions in Cone-Beam Computed Tomographic Images. J. Am. Dent. Assoc..

[54] Setzer F.C., Shi K.J., Zhang Z., Yan H., Yoon H., Mupparapu M., Li J. (2020). Artificial Intelligence for the Computer-Aided Detection of Periapical Lesions in Cone-Beam Computed Tomographic Images. J. Endod..

[55] Fu W.T., Zhu Q.K., Li N., Wang Y.Q., Deng S.L., Chen H.P., Shen J., Meng L.Y., Bian Z. (2024). Clinically Oriented CBCT Periapical Lesion Evaluation via 3D CNN Algorithm. J. Dent. Res..

[56] Kirnbauer B., Hadzic A., Jakse N., Bischof H., Stern D. (2022). Automatic Detection of Periapical Osteolytic Lesions on Cone-Beam Computed Tomography Using Deep Convolutional Neuronal Networks. J. Endod..

[57] Zheng Z., Yan H., Setzer F.C., Shi K.J., Mupparapu M., Li J. (2021). Anatomically Constrained Deep Learning for Automating Dental CBCT Segmentation and Lesion Detection. IEEE Trans. Autom. Sci. Eng..

[58] Li S., Liu J., Zhou Z., Zhou Z., Wu X., Li Y., Wang S., Liao W., Ying S., Zhao Z. (2022). Artificial Intelligence for Caries and Periapical Periodontitis Detection. J. Dent..

[59] Ramezanzade S., Laurentiu T., Bakhshandah A., Ibragimov B., Kvist T., Bjørndal L., Bjørndal L., Dawson V.S., Fransson H., Frisk F. (2023). The Efficiency of Artificial Intelligence Methods for Finding Radiographic Features in Different Endodontic Treatments—A Systematic Review. Acta Odontol. Scand..

[60] Silva V.K.S., Vieira W.A., Bernardino Í.M., Travençolo B.A.N., Bittencourt M.A.V., Blumenberg C., Paranhos L.R., Galvão H.C. (2020). Accuracy of Computer-Assisted Image Analysis in the Diagnosis of Maxillofacial Radiolucent Lesions: A Systematic Review and Meta-Analysis. Dentomaxillofacial Radiol..

[61] Badr F.F., Jadu F.M. (2022). Performance of Artificial Intelligence Using Oral and Maxillofacial CBCT Images: A Systematic Review and Meta-Analysis. Niger. J. Clin. Pract..

[62] Sadr S., Mohammad-Rahimi H., Motamedian S.R., Zahedrozegar S., Motie P., Vinayahalingam S., Dianat O., Nosrat A. (2023). Deep Learning for Detection of Periapical Radiolucent Lesions: A Systematic Review and Meta-Analysis of Diagnostic Test Accuracy. J. Endod..

[63] Abesi F., Maleki M., Zamani M. (2023). Diagnostic Performance of Artificial Intelligence Using Cone-Beam Computed Tomography Imaging of the Oral and Maxillofacial Region: A Scoping Review and Meta-Analysis. Imaging Sci. Dent..

[64] Mohammad-Rahimi H., Motamedian S.R., Rohban M.H., Krois J., Uribe S.E., Mahmoudinia E., Rokhshad R., Nadimi M., Schwendicke F. (2022). Deep Learning for Caries Detection: A Systematic Review. J. Dent..

[65] Ezhov M., Gusarev M., Golitsyna M., Yates J.M., Kushnerev E., Tamimi D., Aksoy S., Shumilov E., Sanders A., Orhan K. (2021). Clinically Applicable Artificial Intelligence System for Dental Diagnosis with CBCT. Sci. Rep..

[66] Venskutonis T., Plotino G., Juodzbalys G., Mickevičiene L. (2014). The Importance of Cone-Beam Computed Tomography in the Management of Endodontic Problems: A Review of the Literature. J. Endod..

